# Complete regression of pulmonary squamous carcinoma in IPF following gemcitabine plus cisplatin: a case report and literature review

**DOI:** 10.1186/s12890-020-1094-1

**Published:** 2020-03-20

**Authors:** Weirong Ma, Hui Li, Zhigang Tian, Shaojin Wang, Xiwei Zheng, Jia Hou

**Affiliations:** 1 Department of Respiratory and Critical Care Medicine, General Hospital of Ningxia Medical University, No.804 Shenglijie, Xingqing District, Yinchuan, 750004 China; 2 Ningxia Medical University, Yinchuan, Ningxia China

**Keywords:** IPF, Lung cancer, NSCLC, Neoplasm regression

## Abstract

**Background:**

Lung cancer is one of the most common co-morbid conditions in patients with idiopathic pulmonary fibrosis (IPF) and negatively affects the prognosis of IPF; Current guidelines for the management of IPF do not give a clear statement on how to manage these patients, and traditional chemotherapy for lung cancer had a limited efficiency rate. Here, we present a rare case of primary lung squamous carcinoma in a patient with IPF whose tumor completely regressed following gemcitabine plus cisplatin therapy; the cancer was no longer detectable after 2 years upon follow-up.

**Case presentation:**

Sixty-seven year-old male patient with IPF was admitted to hospital due to acute onset hemoptysis. In addition to a definite usual interstitial pneumonia (UIP) pattern, a chest CT scan showed a non-enhancing nodular opacity in the right upper lobe and an enhancing nodule in the right lower lobe. Bronchoscopic biopsy of the nodule in the right lower lobe revealed squamous lung cancer. After 2 cycles of chemotherapy with gemcitabine and cisplatin, the tumor in the right lower lobe was no longer detectable after 2 years of follow-up; however, the nodule in the right upper lobe had increased significantly. Finally, *Mycobacterium tuberculosis* (MTB) was cultured from the bronchoalveolar (BAL) sample submitted at the last evaluation, and the patient was confirmed to have active pulmonary TB.

**Conclusion:**

We report the first documented case of complete pulmonary squamous carcinoma regression in IPF following gemcitabine plus cisplatin. Traditional chemotherapy is considered inadequate to cause the resulting regression of the tumor. The concomitant active pulmonary tuberculosis possibly underlies the mechanism.

## Background

Idiopathic pulmonary fibrosis (IPF) is a progressive interstitial lung disease with an undefined etiology and poor prognosis. The prevalence of lung cancer is much higher in IPF patients than in the general population, and lung cancer significantly worsens the prognosis of IPF [1]. However, it is controversial whether IPF patients with lung cancer are potential candidates for radiation therapy or chemotherapy because such procedures, including chemotherapy, biopsies and surgery, might induce acute exacerbations and increase the risk of death [2–4]. We present a rare case of primary lung squamous carcinoma in a patient with IPF whose tumor completely regressed following gemcitabine plus cisplatin therapy. The cancer was no longer detectable and the underlying IPF also remained stable after 2 years of follow-up. Interestingly, while the tumor in the right lower lobe regressed, tuberculosis in the right upper lobe increased significantly, suggesting active tuberculosis possibly underlies the regression of lung cancer.

## Case presentation

**Fig. 1:**
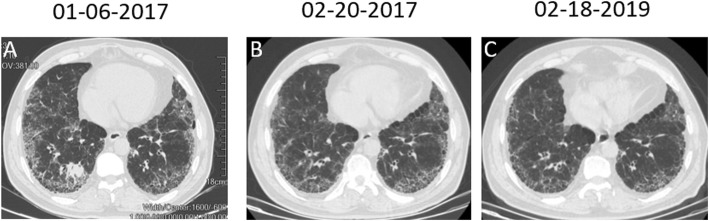
Serial chest computed tomography (CT) scans showing complete regression of the tumor. **a** The chest CT scan at first presentation revealed a tumor in the right lower lobe. **b** The follow-up chest radiograph revealed complete regression of the tumor 1 month after the first cycle of chemotherapy. **c** Complete disappearance of the tumor 2 years after the first presentation

**Fig. 2:**
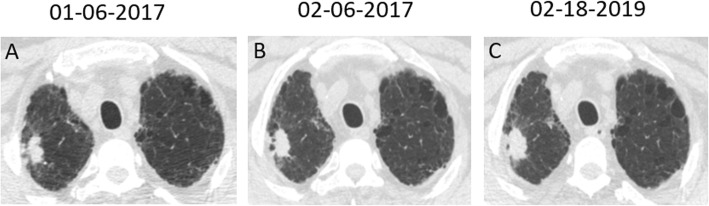
Serial chest CT scan showing enlargement of nodular opacities in the right upper lobe. *Mycobacterium tuberculosis* was cultured from the sputum sample submitted at the last evaluation. He was diagnosed as having pulmonary TB

**Fig. 3:**
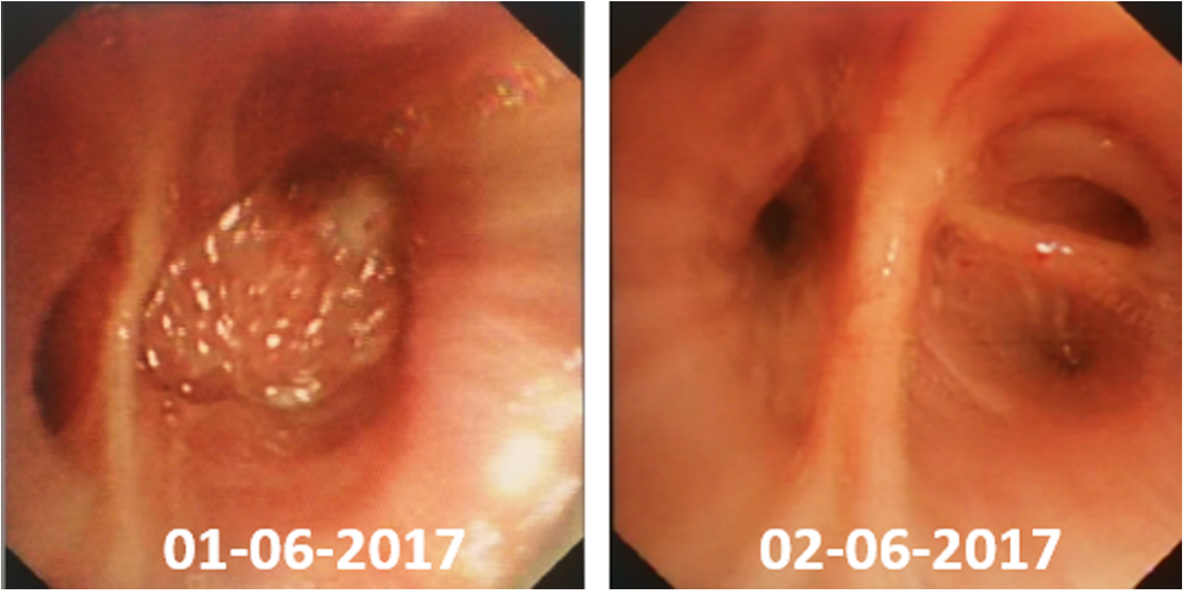
Serial bronchoscopy showed complete regression of the intrabronchial neoplasm in the subsegmental posterior basal segment of the right lower lobe

**Fig. 4:**
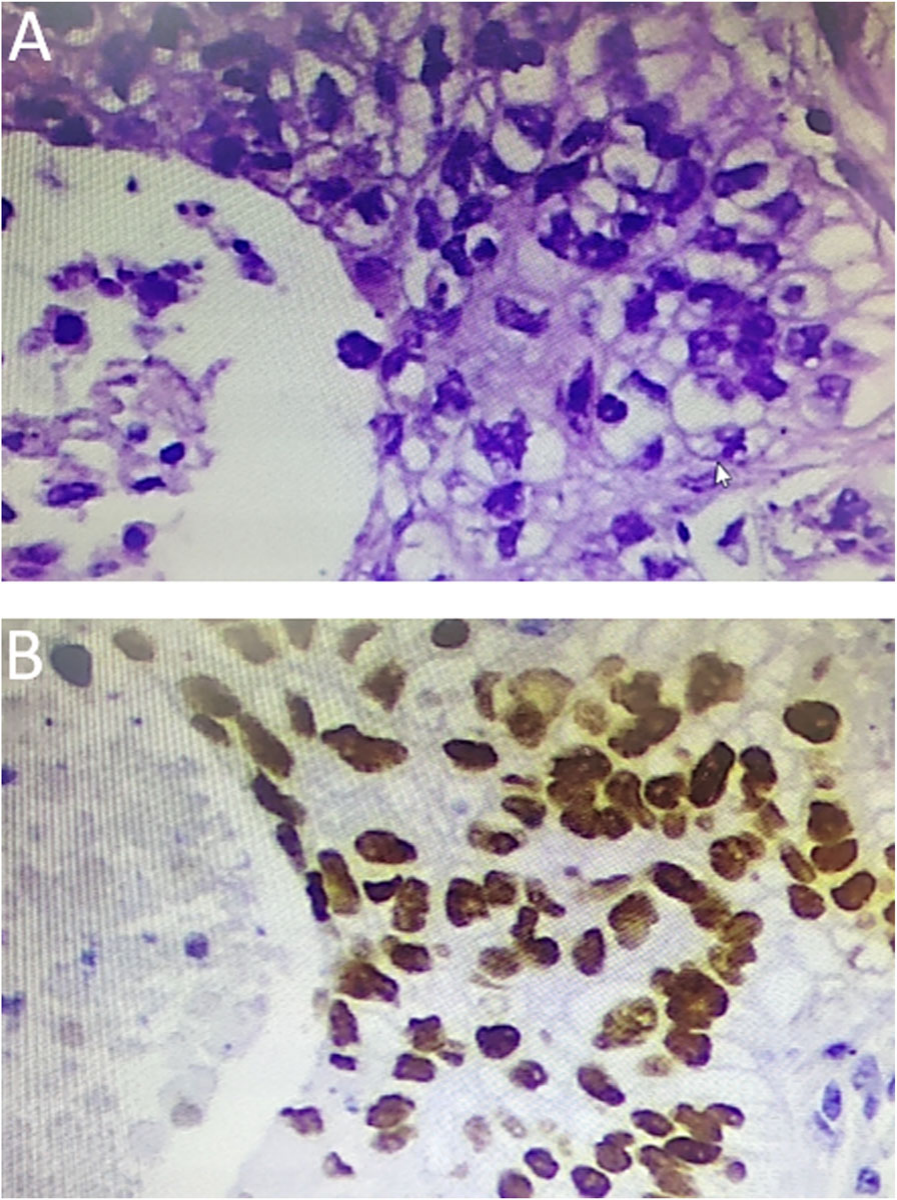
**a** Bronchoscopic biopsy specimen from the right lower showing poorly differentiated non-small-cell lung cancer, specifically squamous cell carcinoma (hematoxylin-eosin, original magnification × 200). **b** Immunohistochemistry revealed that the tumor cells were positive for p40 and p63 expression. Immunohistochemically defining the tumor as a squamous cell carcinoma (× 200)

A 67-year-old man presented for evaluation of acute-onset hemoptysis. He had a cough and mild dyspnea for the past 7 years. He had a 40 pack-year history of smoking and no occupational exposure. Type 2 diabetes was diagnosed > 13 years ago. High-resolution computer tomography (HRCT) demonstrated a definite usual interstitial pneumonia (UIP) pattern with extensive traction bronchiectasis and peripheral honeycombing which were bibasilar-predominant (Fig. 1). Based on the clinical and radiological features, the patient was diagnosed with IPF. Thoracic HRCT also revealed a non-enhancing nodular opacity in the right upper lobe, suggesting pulmonary tuberculosis, and a 25-mm contrast-enhancing nodule in the right lower lobe (Fig. 2). A neoplasm was demonstrated in the sub-segmental posterior basal segment of the right lower lobe with bronchoscopy (Fig. 3). The tumor was biopsied and the patient was pathologically diagnosed with pulmonary squamous lung cancer (Fig. 4). Both *Mycobacterium tuberculosis* (MTB) and a cytological examination assay were negative from the bronchoalveolar (BAL) sample of the right upper lobe. Because there was no evidence of metastatic cancer, the patient was clinically staged as T_1c_N_0_M_0_.

Considering treatments for lung cancer such as surgery or radiation might potentially induce acute exacerbations and even increase the risk of death, the patient was finally treated with gemcitabine (1000 mg/m^2^, days 1 and 8) and cisplatin (75 mg/m^2^, day 1) every 3 weeks, for 6 cycles since January 2017. One month later, after the first cycle of chemotherapy in February 2017, both CT scan and bronchoscopy showed that the pulmonary solitary nodule in the right lower lobe had disappeared entirely (Figs. 1 and 2). After 6 cycles, maintenance chemotherapy with gemcitabine (1000 mg/m^2^, days 1 and 8) was given alone every 3 months for 3 cycles. Subsequently, the tumor was no longer detectable until December 2019 and stable IPF was achieved (Fig. 1). Despite complete regression of lung cancer in the right lower lobe, a follow-up chest CT scan also revealed an increase in the size of the nodule in the right upper lobe (Fig. 2). Finally, *Mycobacterium tuberculosis* (MTB) was cultured and the GeneXpert MTB assay was positive from the bronchoalveolar (BAL) sample of the right upper lobe submitted in January 2019, and he was confirmed to have active pulmonary TB. Once a diagnosis of TB was made, the patient was started on the first-line anti-TB treatment regimen (2HRZE/4HR) for 6 months. With treatment, the nodule in the right upper lobe decreased in size.

## Discussion and conclusions

IPF is a chronic fibrotic lung disease with an undefined etiopathogenesis and a high mortality rate. Although it is difficult to predict the clinical course of the disease, IPF usually involves progressive deterioration with a median survival time ranging from 2.5–3.5 years after diagnosis [5]. Patients with IPF are known to have associated comorbid conditions, which might adversely affect exercise tolerance, quality of life and survival [6]. Lung cancer is one of the most common comorbid condition in IPF. The risk of having lung cancer is much higher in IPF patients than in the general population, and lung cancer can potentially shorten the survival of patients with IPF [1, 7]. Optimal identification and treatment of lung cancer are essential to optimize patient outcomes; however, many diagnostic or therapeutic procedures including pharmacological treatment, biopsy, surgery or radiation might induce acute exacerbations and significantly increase the mortality, making the diagnosis and treatment of lung cancer in IPF patients a challenging task [2, 4]. In addition, current guidelines for the management of IPF do not give a defined statement regarding management of these patients whose prognosis is negatively influenced by lung cancer [8, 9].

Currently, lung cancer is often managed with multidisciplinary treatment strategies, of which chemotherapy remains the basic approach. Gemcitabine plus cisplatin chemotherapy is still considered standard first-line treatment in patients with advanced squamous lung cancer or intolerant to a surgery [10]. Gemcitabine and cisplatin combination therapy can improve the quality of life and extend survival for patients with non-small cell lung cancer (NSCLC) [11, 12]; however, only a small number of cancer patients would benefit from gemcitabine and cisplatin combination therapy. The efficiency rate is only 20–40%, and the median length of survival is 8–10 months, and the 5-year survival rate is < 15% [12–15]. In the present case, gemcitabine plus cisplatin was selected as first-line chemotherapy, and when a complete regression (CR) was achieved, gemcitabine alone was continued. Minimal side effects were observed throughout the course of treatment, and more importantly, the squamous tumor entirely disappeared after 2 cycles of chemotherapy. This phenomenon is extremely rare and beyond our expectations.

In contrast, spontaneous regression (SR) should be considered, although SR is an exceptionally uncommon phenomenon, especially for lung cancer. According to the generally accepted definition, SR of cancer refers to a partial or complete disappearance of a malignant tumor without any treatment or with therapy that is considered inadequate to cause the resulting regression [16, 17]. We do not believe that gemcitabine plus cisplatin led to complete disappearance of squamous lung cancer. Our case can be classified as a complete SR in agreement with the definition of SR; however, it would be extremely difficult to distinguish therapy-induced remission from inadequate therapy coincidental to SR in some cases.

SR is regarded as a rare occurrence; approximately 20 cases are reported in the world literature annually but estimates of the SR incidence in the real world vary widely. Everson and Cole [16]. reviewed all cases of SR from 1900 to 1965, with an estimated incidence of 1/60,000–100,000 cancer patients. Challis et al. [18]. reviewed 741 cases of SR of malignant diseases from 1900 to 1987. Of all individuals, approximately 60% were still alive > 1 year after the regression occurred, while > 25% were still alive after 5 years [18]. The malignant diseases in which SR are most frequently reported include renal cell carcinoma, melanoma, and neuroblastoma [17]. SR of lung carcinoma is a particularly rare occurrence [19, 20], with 18 cases reported in the English literature (11 with NSCLC and 7 with SCLC). Currently, only one case has been reported to involve SR of lung cancer in IPF [21].

Although the exact mechanism underlying SR of cancer is entirely speculative, an immunologic response by multiple conditions, such as infection, necrosis, or operative trauma, might be the cause of SR of lung cancer. Other possible mechanisms include destruction of the feeding artery of the tumor during the biopsy, inhibition of angiogenesis, increased apoptosis, or differentiation induced by causative factors [22, 23]. The co-existence of active pulmonary TB with tumor in our case suggest that the TB infection had a potential role underlying lung cancer regression. The mutual influence between TB and lung cancer has been well-described in many studies but remains controversial. Pre-existing TB predisposes the individual to lung cancer; lung cancer induces re-activation of TB [24]. However, it has been investigated in earlier studies that regression and remission of tumors may occur in patients with a co-existing TB infection [25]. There might be some relationship between the immune activation to TB and lung cancer. Interestingly, there is accumulating evidence that the Th1 response, which is induced by TB infection or BCG vaccination, mediate protection against lung cancer, which may be the reason why BCG can be used as a non-specific immune stimulant to treat malignancies. In a more recent retrospective study [26], NSCLC patients with active pulmonary TB have better survival outcomes than patients without TB, especially squamous cell carcinoma. Active T lymphocyte immunity in tumors may be the underlying mechanism. Based on the above, one possible explanation for our case is that co-existing TB stimulates the immune system and shifts towards Th1 immune responses against tumors. On the other hand, the early stage of the tumor might also be a potential factor of good response to the chemotherapy.

In conclusion, complete regression of squamous lung cancer following chemotherapy is an extremely rare but spectacular event, and it is even rarer in the context of IPF. The precise mechanism of this phenomenon remains a mystery, and no specific factor appears to be responsible for tumor regression. Additional research is needed to explain the possible mechanisms by which this occurs. Knowledge of these mechanisms may help elucidate the nature of lung cancer and disease management.

## Data Availability

All data discussed in the manuscript are included within this published article.

## Abbreviations

IPF: Idiopathic pulmonary fibrosis
NSCLC: Non-small cell lung cancer
SCLC: Small cell lung cancer
SR: Spontaneous regression
TB: Tuberculosis
UIP: Usual interstitial pneumonitis
